# Antimicrobial Susceptibility Profiles of Commensal *Escherichia coli* Isolates from Chickens in Hungarian Poultry Farms Between 2022 and 2023

**DOI:** 10.3390/antibiotics13121175

**Published:** 2024-12-04

**Authors:** Ádám Kerek, Ábel Szabó, Ákos Jerzsele

**Affiliations:** 1 Department of Pharmacology and Toxicology, University of Veterinary Medicine, István utca 2, 1078 Budapest, Hungary; szabo.abel@student.univet.hu (Á.S.); jerzsele.akos@univet.hu (Á.J.); 2 National Laboratory of Infectious Animal Diseases, Antimicrobial Resistance, Veterinary Public Health and Food Chain Safety, University of Veterinary Medicine, 1078 Budapest, Hungary

**Keywords:** *Escherichia coli*, antimicrobial resistance, minimum inhibitory concentration, MIC, poultry, chickens, Hungary

## Abstract

**Background:** Widespread use of antibiotics has led to a global increase in resistance. The *Escherichia coli* bacterium is a facultative pathogen that often develops antibiotic resistance and is easily transmitted, not only in animal health but also in public health. Within the poultry sector, domestic fowl is widespread and one of the most dynamically growing sectors, which is why regular, extensive monitoring is crucial. Among economically important livestock, poultry as a major source of animal protein for humans is a frequent carrier of *Escherichia coli*, also with sporadically detected clinical disease. **Methods:** Our research evaluates the susceptibility of commensal *Escherichia coli* strains, isolated from large domestic fowl flocks in Hungary, to antibiotics of animal and public health importance, by determining the minimum inhibitory concentration value. **Results**: A total of 410 isolates were tested, with the highest level of resistance being found for florfenicol (62.7%). Particularly alarming are the resistance rates to enrofloxacin (52.9%), colistin (30.7%), and ceftriaxone (23.9%). We also found a resistance of 56.1% to amoxicillin and 22.2% to amoxicillin-clavulanic acid, which suggests that the majority of strains are β-lactamase-producing. When compared with the national human resistance data, we found with similar values for amoxicillin and amoxicillin-clavulanic acid, but the resistance rates of aminoglycosides, fluoroquinolones, and potency sulfonamide were worse in animal health. **Conclusions:** In conclusion, our results suggest that periodic surveys should be carried out and that long-term trends can be established that allow the monitoring of resistance patterns over time. For multidrug-resistant strains, new generation sequencing can be used to investigate the genetic background of resistance.

## 1. Introduction

Poultry meat and eggs are undoubtedly a main source of protein for the world’s rapidly growing population, with low production costs and no religious restrictions on consumption. As a result, the poultry sector is one of the fastest growing livestock sectors worldwide. According to the World Food and Agriculture Organization (FAO), global poultry meat production in 2020 was 337 million tons, an increase of 45% compared to that in 2000, and more than half of this was accounted for by domestic chickens [1]. In the European Union, around 400 million laying hens are produced each year, with Spain accounting for around 10% of its egg production [2].

The poultry industry is one of the largest antibiotic-using sectors [3,4]. The bacterium *Escherichia coli* (*E. coli*) is a Gram-negative bacterium that is a common member of the gut microbiome of humans and animals. It is generally commensal, but some strains can cause clinical diseases, usually in the form of diarrheal pathogens and less commonly as systemic infections, such as the separate group of avian pathogenic (APEC) strains [5]. Especially concerning are human infections caused by the consumption of food of animal origin, which can lead to the development of diarrheal diseases.

*E. coli* is classified into five pathotypes, and the most important of these are the shiga toxin-producing *E. coli* (STEC) strains, of which enterohaemorrhagic strains (EHEC) are a subgroup [6]. Human infections caused by STEC strains are sporadic and associated with specific outbreaks. In 2015, 5901 human infections caused by STEC strains were confirmed in the European Union (EU), although poultry were found to be asymptomatic carriers of these strains [7]. *E. coli* is a commensal indicator bacterium used to monitor AMR in public health as a potential threat from food-producing animals [8,9].

Although commensal *E. coli* strains are of no direct clinical significance, they could potentially transmit resistance genes to pathogenic strains. A study from the Netherlands in 2022 found that the resistance profile of pathogenic *E. coli* strains isolated from broiler chickens and clinical cases was very similar [10]. In *E. coli*, transcription of the most common plasmid-mediated resistance genes has been observed in genes encoding beta-lactamases, efflux pumps, aminoglycoside phosphorylases and hydrolases, and chloramphenicol transacetylase [11,12].

The very high antimicrobial resistance gene prevalence observed in day-old chicks hatching from eggs [13] is thought to be derived from the environment and the vertically transmitted gut microbiota of birds [14]. Horizontal gene flow primarily leads to the widespread emergence of antimicrobial resistance on farms and in the environment [11], but *E. coli* can also develop resistance through mutations and horizontal gene transfer [15].

A major problem is the worldwide spread of the so-called New Delhi metallo-β-lactamase-producing strains, which can hydrolyze penicillins, cephalosporins, and carbapenems but not monobactams. β-lactamase inhibitors are also unable to inhibit its function [16]. In the global surveillance program, 19.4% of Enterobacteriaceae strains tested positive for carbapenemase [17]; however, in China, this figure had risen to 31% [18], while in Europe, the rate for *E. coli* bacteria was 10.3% [19]. According to a report by the European Centre for Disease Prevention and Control, their prevalence in European countries increased almost fivefold between 2017 and 2021 [20].

The avian pathogen *E. coli* can cause pericarditis, perihepatitis, septicemia, intestinal inflammation, arthritis, and cellulitis [21,22], and in laying hens, it can contribute to a qualitative and quantitative reduction in egg production through salpingitis [23]. *E. coli* bacteria are a substantial reservoir of antimicrobial resistance genes, as well as genes encoding virulence factors, and this poses a particularly serious threat in the case of extra-intestinal pathogenic *E. coli* (ExPEC), including uropathogenic *E. coli* (UPEC) and neonatal meningitis *E. coli* (NMEC) strains [24,25]. Colistin is a critically important antibiotic; however, it was not included in Europe’s routine AMR-monitoring panel until 2014, resulting in a limited selection of available publications in recent years [26]. European monitoring reports from 2014 and 2016 show a low prevalence of resistance in broiler chickens, with 3.9% observed in chicken meat and less than 1% in Scandinavian countries. However, higher prevalence rates were reported in Germany, Italy, Switzerland, and Portugal [27]. In 2022, the EU mandated AMR monitoring for *E. coli* strains isolated from the cecal content of broiler chickens. High resistance rates were observed against ciprofloxacin (51.4%) and nalidixic acid (48%). Analyzing resistance trends over time (2009–2022), significant decreases (*p* < 0.05) were observed in resistance to ampicillin, ciprofloxacin, cefotaxime, and tetracycline in most member states. Exceptions included Norway and Cyprus, where increasing trends were reported [28].

Due to the high morbidity and mortality of these infections, antibiotics are usually given per os to chickens [29], but in developing countries, they are still used for prophylactic treatment and as growth promoters [30]. This is concerning as resistance to third-generation cephalosporins [31] and colistin [32] becomes more widespread. In the future, antibiotic-induced selection pressure should be reduced, thereby also reducing the number of resistant strains. Partial or total replacement could be possible with the use of various plant essential oils [33], plant extracts [34,35], or antimicrobial peptides [36]. The active components of propolis are mostly flavonoids, with different degrees of synergistic action that have been shown to have antimicrobial efficacy [37,38,39]. As the poultry industry is one of the biggest users of antibiotics, it would be particularly important to use less antibiotics and to use them more responsibly in this sector [40]. Preservation of the efficacy of active substances is possible if they are used in a correct way, based on pharmacokinetic/pharmacodynamic studies [41].

The *E. coli* can be classified into phylogenetic groups A, B1, B2, and D [42,43], of which groups A and B1 usually include multidrug-resistant strains [44], and they are found not only in poultry, but also in humans [45]. However, certain studies have failed to show a link between phylogenetic group- and multidrug-resistance [46,47]. Groups B2 and D mainly contain extraintestinal strains isolated from human and avian pathogens [45,48]. Cross-contamination occurs most commonly during butchering, and chicken meat is an excellent substrate for commensal microbes such as *E. coli*. The risk posed by commensal microbes is increased by the presence of antimicrobial resistance genes and the presence of shiga toxin-producing strains, which are capable of causing diseases in humans [49,50]. Furthermore, the biofilms produced by *E. coli* also pose a major challenge as they allow the bacteria to adhere to the surfaces of objects and persist for a long time [51,52]. One slaughterhouse monitoring study identified *E. coli* strains isolated from chicken meat as being 56.6% A, 28.3% B1, 6.0% B2, and 9.1% D phylogenetic groups [53].

The level of aminoglycoside resistance in chickens is particularly high. Resistance rates of 62% and 64% for kanamycin and streptomycin, respectively, were described as early as 1985 [54]. However, a longitudinal study of *E. coli* resistance from 1998 to 2016 in the Netherlands showed that resistance to most of the tested active substances started to decrease in 2010. It is probable that this is due to ceftiofur, a third-generation cephalosporin antibiotic, being used as a preventive agent in day-old chicks and hatcheries in the Netherlands until 2010 [8].

In a series of studies carried out between 2019 and 2020, 27 EU Member States and five non-EU countries reported data on the antimicrobial (AMR) status of *E. coli* strains isolated from the cecal contents of slaughtered food-producing animals. Among the studies, broiler chicken samples were tested for the presence of strains carrying genes responsible for the production of extended-spectrum beta-lactamase (ESBL), *ampC* genotype, and carbapenemase genes. It was found that the ESBL rate was 1%, and the presence of the *ampC* gene was 0.3% [55]. In addition, the studies showed that multidrug-resistant strains occurred at a prevalence of 38.7%, resistance to fluoroquinolones was critical (60.7% for ciprofloxacin and 51.8% for nalidixic acid in broilers), and resistance to chloramphenicol and gentamicin was also noted as high to very high [55]. However, in the same studies, resistance to colistin was barely observed. Similarly, for ceftazidime and cefotaxime, resistances of just 0.5% and 0.4% were described [55]. In Hungary, following the swine sector, the poultry industry is the second-largest consumer of antibiotics, accounting for 23.78% of the total antibiotics used. In 2022, the most frequently used antibiotic in broiler chickens was amoxicillin (1.937 tons), followed by enrofloxacin (0.865 tons) and doxycycline (0.500 tons) [56].

The zoonotic potential of *E. coli* strains isolated from poultry origin is concerning, as their virulence profiles and phylogenetics are often similar to those found outside the intestinal tract in humans [57]. Alarmingly, it has been shown that cephalosporin resistance genes can be transferred from *E. coli* found on chicken meat to *E. coli* in humans, which can cause difficulties in the management of clinical infections [58]. Therefore, continuous testing of commensal *E. coli* strains is particularly important to monitor the status of antimicrobial resistance. The strains evaluated in the national monitoring program of each country should be regularly checked in order to reveal trends which may be of significance for public health.

## 2. Results

### 2.1. Regional Distribution and Origin of the Samples

We performed susceptibility testing of a total of 410 *E. coli* strains, isolated from domestic chickens, to 15 different antibiotic substances of animal and public health importance. In terms of the regional distribution of the samples, 9.1% were from Nyugat-Dunántúl, 11.3% from Közép-Magyarország, 16.5% from Közép-Dunántúl, 16.8% from Észak-Magyarország, 17.3% from Észak-Alföld, 18.1% from Dél-Dunántúl, and 10.9% from Dél-Alföld. Of the samples, 41.1% were taken from the respiratory system, and 58.9% were from the cloaca. Considering the type of chickens, 45.1% of the samples came from meat flocks, 23.9% from breeding flocks, and 31.0% from egg flocks. In terms of age, 54.9% of the samples were isolated from adult birds and 45.1% from juveniles. In addition, regarding flock size, 43.2% of the samples were from small (5001–50,000), 32.2% from medium (50,001–100,000) and 24.6% from large (>100,001) flocks.

### 2.2. Antimicrobial Susceptibility Testing

We determined MIC values for a total of 11 agents with clinical breakpoints; these breakpoints were defined on the basis of Clinical Laboratory Standard Institute (CLSI); and literature data were used for amoxicillin-clavulanic acid [59], neomycin [60], spectinomycin [59], and colistin [10].

**Figure 1:**
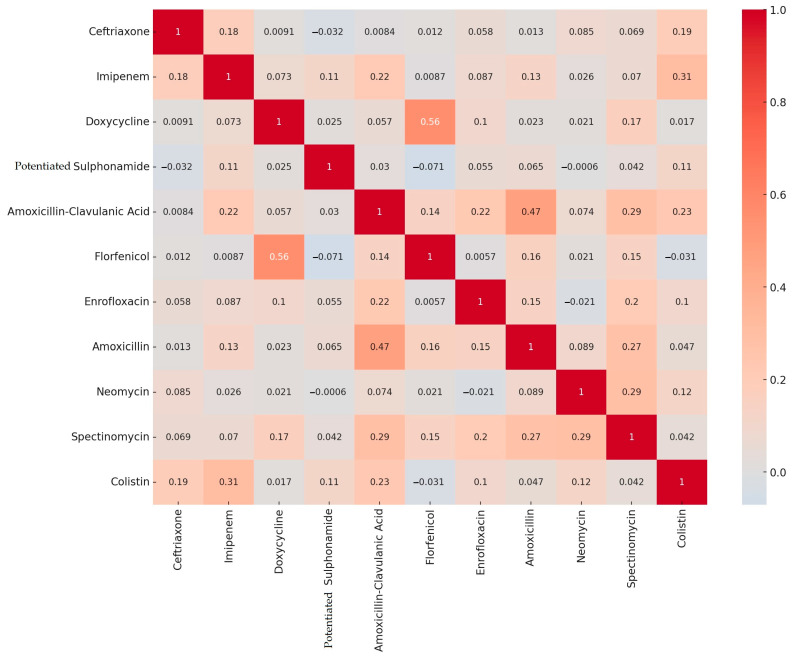
A correlation study of antimicrobial resistance to individual substances, based on the results of *Escherichia coli* (n = 410) samples isolated from domestic chickens.

The breakpoints were used to determine the degree of resistance for each active substance, and a correlation study was performed for each active substance (Figure 1). Positive correlation values indicate that the resistance rates of the antibiotics in question increase or decrease together. Negative correlation values indicate that the resistance to one antibiotic is increasing while that of the other is decreasing.

There is a relatively high positive correlation between florfenicol and doxycycline (0.56), as well as between amoxicillin and amoxicillin-clavulanic acid (0.47). However, there is a weak negative correlation between florfenicol and potentiated sulfonamide (−0.071), as well as between florfenicol and colistin (−0.031).

**Figure 2:**
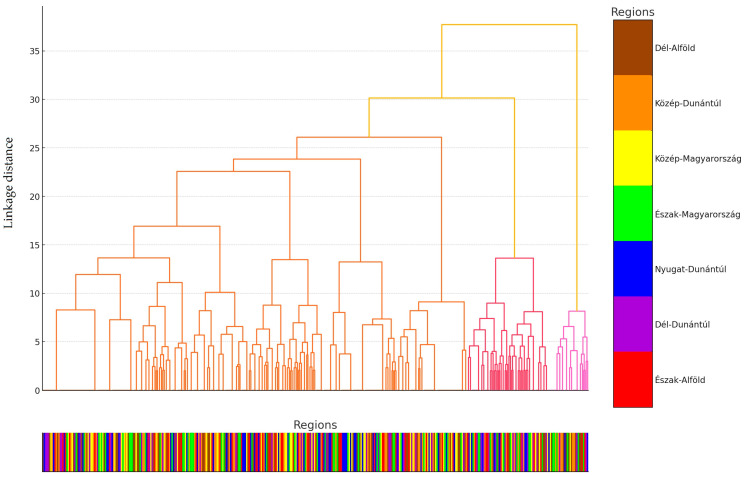
Cluster analysis of *Escherichia coli* strains (n = 410) isolated from domestic chickens based on the antibiotic resistance profile of the samples.

We performed a cluster analysis of the samples (Figure 2). Cluster analysis is a statistical method that aims to organize the data into homogeneous groups, called clusters. In the analysis, data points are split so that elements in one cluster are similar to each other, and elements in different clusters are different from each other. To make the number of sample elements more transparent, each sample is assigned a regional origin, and on the horizontal axis of the graph, after color-coding the regions, a straight horizontal bar of lines of the corresponding color represents the samples.

**Figure 3:**
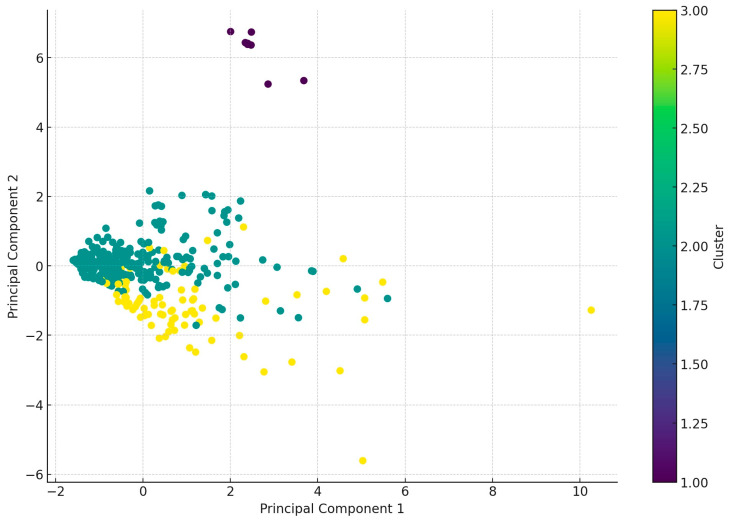
The analysis performed using principal component analysis (PCA) in conjunction with cluster analysis grouped the samples into three main clusters based on their antimicrobial resistance patterns (purple—1, green—2, yellow—3).

Using principal component analysis (PCA), the samples were grouped into three main clusters (Figure 3). Cluster 1 included 9 samples, Cluster 2 included 315 samples, and Cluster 3 included 86 samples. All samples in Cluster 1 were from the Észak-Magyarország region. The samples in Cluster 2 showed an even distribution, with the exception of the Közép-Magyarország region, where the sample size was lower. In the case of Cluster 3, the sample size was larger in the Közép-Magyarország, Észak-Alföld, and Észak-Magyarország regions.

PCA is a dimensionality reduction technique aimed at reducing the dimensionality of data while preserving its variance. PCA creates new variables (principal components) that are linear combinations of the original variables. The individual clusters reflect the similarity of samples based on antibiotic resistance, which can be useful for further analysis and decision-making.

We performed the statistical analysis of the samples using a two-sample *t*-test, based on the sample source (respiratory tract, cloaca) (Table 1). It is clear that the sample source had minimal influence on the level of resistance, with significant differences observed only for ceftriaxone (*p* = 0.0327) and spectinomycin (*p* = 0.0494).

In addition to the type of sample, we performed a comparison based on the utilization direction (laying, meat, or breeding), which is presented in Table 2. The results reflect that the utilization direction plays a decisive role in the resistance patterns, with significant differences observed in the level of resistance between meat-producing and breeding stocks for most active substances. The strongest difference was observed between the results for amoxicillin and amoxicillin-clavulanic acid (*p* < 0.0001). This is significant due to the widespread use of amoxicillin within the poultry sector. For amoxicillin-clavulanic acid, the combination of active substances is not authorized in the absence of a maximum residue level (MRL) value. However, the results of in vitro studies show a significant production of β-lactamase by the strains.

We also examined the differences between age groups (Table 3), and significant differences were observed between juvenile and adult animals, with significant differences found for seven out of the eleven antibiotics. Age did not affect the levels of resistance for doxycycline, potentiated sulfonamides, florfenicol, and colistin. Again, the most notable difference was also observed for amoxicillin and amoxicillin-clavulanic acid (*p* < 0.0001).

Finally, we examined the differences between flock sizes (Table 4). The resistance profiles between small (5001–50,000) and medium-sized (50,001–100,000) farms showed the least difference, with a significant difference observed only for colistin (*p* = 0.0027). Significantly differently results were found for several active substances between medium and large (>100,001) farms, but the largest difference was observed between small and large-sized farms.

MIC_50_ and MIC_90_ values also assist in monitoring antibiotic resistance trends and in the development of new antibiotics. These values are often used in epidemiological studies to compare antimicrobial resistance patterns across different geographical areas, time periods, or pathogens. The MIC_50_ is the minimum inhibitory concentration at which 50% of the given bacterial population is susceptible, meaning that this antibiotic concentration inhibits growth in half of the bacteria. Thus, the MIC_50_ is a median value indicating that half of the bacteria are more susceptible or equally susceptible to this concentration. The MIC_90_ is the minimum inhibitory concentration at which 90% of the given bacterial population is susceptible, meaning that this antibiotic concentration inhibits growth in 90% of the bacteria. The MIC_90_ is an upper quantile value indicating that a large majority of the bacteria are susceptible to this concentration. The MIC_50_ and MIC_90_ values make it easy and quick to interpret the effectiveness of an antibiotic against a specific bacterial population.

We created a frequency table from the determined MIC values (Table 5), which includes the breakpoints along with the calculated MIC_50_ and MIC_90_ values, as well as the ECOFF values provided by EUCAST. The ECOFF value is the highest MIC value that separates the wild-type population (those strains that do not possess acquired resistance mechanisms) from strains likely carrying resistance mechanisms. When determining ECOFF values, the MIC distribution of wild-type bacteria is considered, and the goal in selecting the value is to ensure complete separation of the wild-type population from the resistant population.

The clinical breakpoint was exceeded for ceftriaxone, imipenem, spectinomycin, doxycycline, colistin, and potentiated sulfonamides at the MIC_50_ value. The MIC_50_ values for amoxicillin-clavulanate, ceftriaxone, imipenem, and colistin were below the ECOFF value. This means that half of the population is presumably wild-type for these agents. The frequency table for agents without established breakpoints is included in Supplementary Table S1.

**Figure 4:**
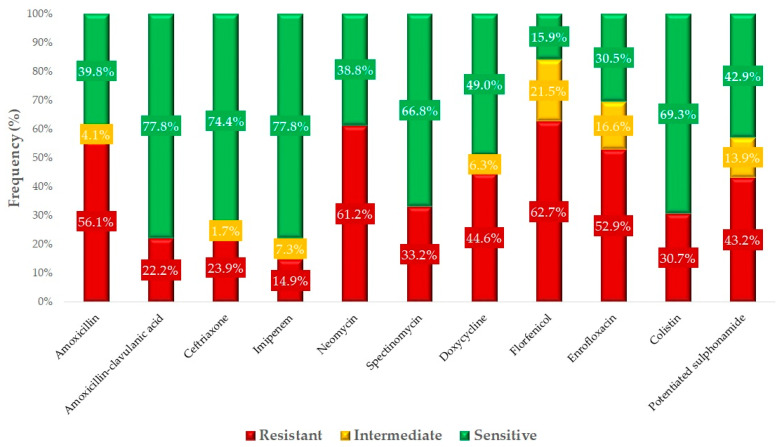
The susceptibility of *Escherichia coli* strains isolated from broilers (n = 410) to antibiotics of animal and public health significance.

Based on the available clinical breakpoints, we determined the antimicrobial susceptibility profile of the individual agents (Figure 4). Among the critically important agents, the resistance level to enrofloxacin is the most severe (52.9%), followed by colistin (30.7%) and ceftriaxone (23.9%). The highest level of resistance was observed against florfenicol (62.7%), closely followed by neomycin (61.2%). The difference in resistance to amoxicillin (56.1%) and amoxicillin-clavulanate (22.2%) indicates that the majority of the strains are β-lactamase-producing strains.

**Figure 5:**
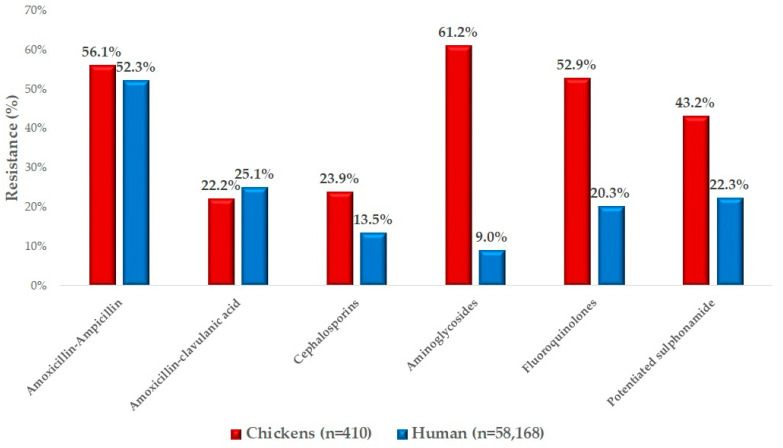
Comparison of *Escherichia coli* strains isolated from broilers and human resistance data for available antibiotics.

We had the opportunity to compare our results with human resistance data (Figure 5). It can be observed that the resistance patterns in animal health and public health were very similar. The results for amoxicillin, a commonly used antibiotic in broilers, are comparable to those for ampicillin used in public health. This was also evident for amoxicillin-clavulanate. However, the situation regarding resistance to cephalosporins (critically important antibiotics) was much worse in broilers (23.9%) compared to the 13.5% resistance rate observed in humans. The resistance levels for aminoglycosides, fluoroquinolones, and potentiated sulfonamides were also significantly worse in animal health.

## 3. Discussion

We examined the susceptibility of a total of 410 isolated *Escherichia coli* strains to 15 antibiotics of veterinary and public health significance, 11 of which had clinical breakpoints. Overall, our samples exhibited higher average resistance values compared to those in the literature. In evaluating our results, we adhered to the CLSI veterinary guidelines, which are widely used globally for establishing clinical breakpoints. This approach ensures the reliability, reproducibility, and comparability of our findings with those of other studies.

The cluster analysis highlights that the differing antibiotic usage practices across livestock farms may influence the clustering patterns. Although this study did not have the opportunity to assess antibiotic usage directly, we are hopeful that in the future, we will find a practical and feasible approach to include these data. Primarily, the significant differences observed in resistance levels between various animal utilization types played a major role in influencing the clustering. To a lesser extent, factors such as the source of the samples and the size of the livestock population also contributed to the clustering patterns.

For amoxicillin, we observed 56.1% resistance, while Hassan et al. reported 32% resistance to ampicillin [61]; Kaushik et al. found 89.4% resistance to penicillin and 80.4% to ampicillin [62]; and Much et al. reported 19% resistance to ampicillin in organic farming and 33.8% in conventional farming [63]. De Jong et al. found resistance rates between 32.7% and 65.3% to ampicillin [64], with Rivera-Gomis et al. reporting 30.8% resistance [65]. The level of resistance observed for amoxicillin in *E. coli* may be associated with its long-standing and widespread use, as well as the fact that members of the Enterobacteriaceae family often produce β-lactamase enzymes, which are responsible for the enzymatic cleavage of this antimicrobial.

For amoxicillin-clavulanic acid, we observed 22.2% resistance, whilst Majewski et al. reported 84.6% resistance to amoxicillin-clavulanic acid [66]. This confirms that most strains produce β-lactamase. While clavulanic acid is not authorized for use in poultry in the absence of an MRL value, monitoring its in vitro effectiveness is important for both animal and public health.

For ceftriaxone, we found 23.9% resistance, similar to Kaushik et al.’s reported 28.2% [62]. However, Mandal et al. described 78.1% resistance to cefotaxime [67]. Ceftriaxone is a critically important antibiotic reserved for hospitalized human patients. Its use is not approved in poultry; nevertheless, resistance to it has recently been widely observed. This resistance is likely explained by cross-resistance developing from the use of other approved antibiotic classes. Investigating the cause of this resistance is certainly warranted in the future.

Resistance to imipenem was 14.9%, which is concerning. Shaib et al. did not detect any resistance [68], while Moffo et al. reported approximately 20% resistance to imipenem [69]. For imipenem, its relatively rapid degradation in aqueous solution is well-known, and due to these stability issues, our results should be interpreted with caution.

For neomycin, we found 61.2% resistance, similar to Majewski et al.’s 84.6% [66]. However, Kaushik et al. reported 13% resistance to gentamicin [68], De Jong et al. reported between 0.9% and 7% [64], and Rivera-Gomis et al. found 12.3% resistance to gentamicin [65]. In our study, the resistance rate to spectinomycin was 33.2%, with Adelowo et al. reporting 47% [70]. The level of resistance to aminoglycosides can be attributed to the extensive use of this class of antibiotics over several decades.

We recorded a resistance rate to doxycycline of 44.6%, with Hassan et al. reporting a similar 44% [61] and Majewski et al. reporting 36% [66]. In contrast, Mandal et al. described 78.1% resistance to tetracycline [67], De Jong et al. found rates between 41.3% and 67.5% [64], and Rivera-Gomis et al. reported 62.1% [65]; meanwhile, Kaushik et al. reported a rate of just 17.4% [62], while Much et al. observed rates of 27.6% and 25.9% in organic and conventional farming, respectively [63]. This is also a class of antibiotics that has been in use for several decades. Due to the overuse of certain agents with poor absorption, it has resulted in significant environmental exposure over time, which has contributed to the widespread development of resistance.

As for florfenicol, we found a resistance rate of 62.7%, whereas Wang et al. reported 0.9% resistance [71] and Adelowo et al. found no resistance [70]. The level of resistance observed against florfenicol is concerning, likely reflecting the broad usage of this agent. Although we lacked specific data on antibiotic use in individual farms due to the non-public nature of these records, which prevented us from drawing direct correlations, it is essential in the future to assess reliable data on antibiotic usage alongside resistance levels, whether at the farm or regional level. For enrofloxacin, we observed 52.9% resistance, higher than that by Majewski et al. (34.6%) [66] and Hassan et al. (32%) [61]. Kaushik et al. reported 6.5% resistance to ciprofloxacin [62], whilst De Jong et al. found rates between 7.3% and 21.3% [64]. Much et al. reported 33.3% and 69.1% resistance to ciprofloxacin in organic and conventional farming, respectively [63]. Enrofloxacin is also a critically important antibiotic, reserved for use in human inpatient care. The high level of resistance observed against enrofloxacin is particularly concerning, especially given its widespread use in the poultry industry. Although enrofloxacin compounds are bitter and water-soluble, poultry’s limited taste sensitivity likely facilitates its usage. From a public health perspective, there are additional concerns, as enrofloxacin can partially metabolize into ciprofloxacin within the body. Since ciprofloxacin is extensively used in human healthcare, resistance to it may be further exacerbated through this indirect exposure.

In our experiments, we found 30.7% resistance to colistin, while Kempf et al. reported 0.6% resistance [26] and Adelowo et al. did not detect any resistant strains [70]. Colistin is frequently used to treat enteric infections caused by *E. coli*. As a critically important antibiotic, its usage should be significantly curtailed to preserve its effectiveness for future generations. The observed resistance to colistin is alarming, and it is essential to investigate the underlying causes of this resistance. This investigation should assess the stability of the compound, along with its binding to organic materials. Additionally, for strains showing resistance, examining the genetic basis of this resistance is warranted.

For potentiated sulfonamides, 43.2% of the strains were resistant, in line with Hassan et al.’s findings of 38% [61], De Jong et al.’s observations of between 27.5% and 49.7% [64], and Majewski et al.’s report of 53.5% resistance [66]. This is also a class of compounds that has been used in veterinary medicine for several decades, which has contributed to the widespread resistance observed against it.

Continuous monitoring of antimicrobial resistance provides valuable insights for legislative bodies, enabling them to track temporal trends and propose measures aimed at promoting appropriate and responsible antibiotic usage. Additionally, it is crucial to emphasize the importance of investigating the underlying genetic causes of resistance, particularly in the case of multidrug-resistant strains. While next-generation sequencing (NGS) remains a costly process and is not yet part of routine daily diagnostics, it offers significant benefits in uncovering underlying mechanisms and establishing causal relationships. This highlights the growing need to integrate genetic analyses alongside phenotypic investigations to enhance our understanding and management of antimicrobial resistance.

In terms of different variables, we found that sample type (respiratory, cloacal) had the least impact on the resistance pattern. This is likely due to the birds’ habit of pecking in environments contaminated with feces, leading to colonization of the pharyngeal mucosa by bacteria ingested from the environment.

More decisive was the type of utilization, with significant differences observed between broiler and breeder stocks. In our previous studies [72], we found similar results, which can be explained by the different rearing times and the varying uses of antibiotics.

When comparing the effects of flock size, the most significant differences were observed between small and large-scale farms, likely due to the different amounts of antibiotics used, technologies applied, and differing precision in dosing. Much et al. also found that resistance was lower in smaller flocks, which they attribute to more accurate dosing. It is likely that the reasons for our findings are similar, with smaller flocks being easier to treat precisely. Based on this, it may be recommended that keeping smaller flocks could be a way to reduce antibiotic resistance *in E. coli* strains [63].

We compared the resistance profile of *E. coli* strains isolated from broilers with the data for *E. coli* resistance in humans. However, a limitation of the comparison is that the available human data were two orders of magnitude larger, as a larger dataset allows for better biological variance and statistical analysis. However, the connection between public health and animal health is undeniable, as *E. coli* is a facultative pathogenic bacterium that can also act as a zoonotic pathogen. Therefore, antimicrobial resistance observed in animal health should always be evaluated in parallel with data from human healthcare to provide a comprehensive understanding of its implications.

Similar results were found for amoxicillin and ampicillin, as well as for amoxicillin-clavulanic acid. In human studies, Carmona-Cartaya et al. reported 68% resistance to ampicillin and 28% resistance to ampicillin-sulbactam [73]; this further indicates that these strains generally produce β-lactamase enzymes. While Carter et al. found 39.2% resistance to ampicillin and 7.6% resistance to amoxicillin-clavulanic acid [74], this similarly suggests the production of β-lactamase enzymes, much like in our case. Enyinnaya et al. reported 100% resistance to ampicillin and 67.8% resistance to amoxicillin-clavulanic acid [75].

For enrofloxacin, we observed 52.9% resistance in poultry, compared to 20.3% in human data. In human studies, Carmona-Cartaya et al. reported 55% resistance to ciprofloxacin [73], while Carter et al. found just 9% [74], and Enyinnaya et al. reported 54.08% resistance [75]. The high enrofloxacin resistance observed in the poultry sector supports the widespread use of this drug, and it is also likely indicative of its frequent use without prior sensitivity testing. When comparing human data, the limitation may lie in the different sample sizes, which were an order of magnitude larger in the human data. However, it is also important to note that human sensitivity tests are based on disk diffusion methods, while we applied the gold-standard microdilution method.

For aminoglycosides, resistance was 61.2% in poultry, while the human data showed only 9% resistance. In human studies, Carter et al. reported 7% resistance to gentamicin [74], while Enyinnaya et al. observed 52% resistance [75]. For potentiated sulfonamides, we observed 43.2% resistance in poultry, while the human data showed 22.3%. In human studies, Carmona-Cartaya et al. reported 1% resistance to potentiated sulfonamides [73]. The overuse of aminoglycosides for several decades is clearly reflected in the very high resistance values observed, as was also the case with sulfonamides.

For cephalosporins, we observed 23.9% resistance, compared to 13.5% in human samples. In human studies, Chua et al. reported 5.7% resistance [76], Carmona-Cartaya et al. found 35% resistance to cefazolin [73], and Carter et al. observed 6% resistance to cephalexin [74]. Although the difference observed in the case of cephalosporins is not significant, considering that their use is not authorized in poultry, the results raise concerns. It is definitely recommended to conduct research aimed at investigating the suspected cross-resistance. Studies targeting this should focus on genetic investigations, induced resistance testing, and transcriptomics.

Correlation analyses highlight the need for more in-depth investigations into resistance patterns. However, as they represent a snapshot of the current state, they are not suitable for drawing far-reaching cause-and-effect conclusions. The observed strong positive correlations may be attributed to the similarity in the mechanisms of action of the antimicrobial agents. Further studies, including genome sequencing as well as pharmacokinetic and pharmacodynamic investigations, are required to elucidate these relationships.

We believe that effectively monitoring resistance trends requires at least annual exploratory surveillance studies. These studies should include, as a first pillar, the examination of commensal strains, while a second pillar would involve incorporating data from strains isolated from clinical cases. Finally, as a third pillar, these findings should be cross-referenced with periodic human resistance data, thereby establishing the One Health approach.

## 4. Material and Methods

### 4.1. The Origins of Poultry Samples and Data on Human Infections

The analyzed strains were collected from large livestock poultry farms in 2022 and 2023, during routine diagnostic examinations of veterinarians. Information available for the samples included the organ (trachea, cloaca), the type of bird (meat, eggs, breeding), the age (young, adult), and the flock size (5001–50,000; 50,001–100,000; >100,001), and based on the location of the flock, the samples were classified into seven administrative regions of Hungary. During the sampling process, the explicit criteria considered were the geographical regions (covering all regions of Hungary) and voluntary participation. During routine diagnostics, veterinarians typically collect cloacal and respiratory samples from live animals. These sample types are preferred because *E. coli* is part of the normal gut microbiota and, due to the animals’ natural pecking behavior, it also colonizes the oropharyngeal cavity. From samples taken during routine diagnostics, strains were isolated on ChromoBio^®^ Coliform (Biolab Zrt., Budapest, Hungary) medium, on which *E. coli* form blue-colored colonies with good growth. The isolated blue colony-forming units were subsequently inoculated onto tryptone soy agar (Biolab Zrt., Budapest, Hungary) to form colony cultures. After collection, the samples were stored in a Microbank™ system (Pro-Lab Diagnostics, Richmond Hill, ON, Canada) at −80 °C.

Human resistance data was provided by the Hungarian National Centre for Public Health and Pharmacy.

### 4.2. Minimum Inhibitory Concentration (MIC) Determination

Phenotypic expression of resistance was assessed by determining minimum inhibitory concentration (MIC) values according to the CLSI methodology [77]. Breakpoints were also defined according to CLSI guidelines [77] and compared with the epidemiological cut-off values (ECOFF) set by the European Committee on Antimicrobial Susceptibility Testing (EUCAST). In the case of neomycin, a breakpoint was found for *E. coli* in a study [60] and for amoxicillin-clavulanic acid [59], spectinomycin [59], and colistin [10] also based on the literature.

After being stored at −80 °C, the bacterial strains were suspended in 3 mL of cation-adjusted Müller-Hinton broth (CAMHB) and incubated for 18–24 h at 37 °C. The studies were performed using 96-hole microtiter plates (VWR International, LLC., Debrecen, Ma-Hungary). All holes except the first column of the working plates were filled with 90 µL CAMHB. The preparation of 1024 µg/mL stock solution data for the test substances (Merck KGaA, Darmstadt, Germany) was performed according to CLSI guidelines [77]. A total of 180 µL of a 512 µg/mL solution diluted in half with CAMHB was spiked into the first column of the working plates and used to prepare a two-fold dilution series. After column 10 of the microtiter plate, the excess 90 µL of solution was discarded, leaving 90 µL of solution in each column. Using a nephelometer (ThermoFisher Scientific, Budapest, Hungary), a bacterial suspension set at 0.5 McFarland was inoculated backwards from column 11 of the microtiter plates at 10 µL/well [77]. The evaluation was performed using Sensititre^TM^ SWINTM automatic MIC reader (ThermoFisher Scientific, Budapest, Hungary) and VIZION system software vs. 3.4 (ThermoFisher Scientific, Budapest, Hungary, 2024). Reference isolate was *Escherichia coli* (ATCC 25922).

Statistical analysis of the data was performed using R program version 4.1.0 [78]. Normal distribution was tested using a Shapiro–Wilk test, data not following normal distribution were further tested by non-parametric tests. The level of resistance of each active substance was assessed using the Kruskal–Wallis test for different aspects [79]. This test does not assume a normal distribution of the data and is suitable for comparing the medians of multiple sample sets, making it ideal for analyzing differences between groups. A further post hoc test was then used to determine the exact associations between the groups. This was done using Mann–Whitney U test [80] and t-test, comparing each type pairwise and then correcting for inflated *p*-values from multiple comparisons using the Bonferroni correction [81]. It should be noted that the Bonferroni correction may increase the chance of a second-order error (failure to detect real differences). Further correlation studies were performed, looking for correlations between the individual active substances, and principal component analysis (PCA) was performed [82], which helps identify similarities or differences between patterns, and then hierarchical cluster analysis was performed using a dendrogram [83]. This figure provides a visual representation of the distances between samples and the hierarchy of clustering.

## 5. Conclusions

Overall, we can conclude that significant resistance was observed in *Escherichia coli* strains against enrofloxacin, due to the widespread use of fluoroquinolones in the poultry sector. The much lower resistance to amoxicillin-clavulanic acid compared to amoxicillin suggests that most strains are β-lactamase producers. Comparing these results with human resistance data follows the One Health approach.

The evolution of resistance, especially against critically important agents such as amoxicillin, supports the necessity of regular monitoring surveys. In the future, periodic surveys could help establish temporal trends. Taking into account the antibiotic use at individual farms and using an adequate sample size to compare results at the regional level could form a solid basis for identifying resistance patterns and predicting future trends. In the case of strains proven to be multidrug-resistant, mapping the genetic background behind the resistance in detail using next-generation sequencing would be worthwhile. Genetic studies could shed light on the regional distribution of multidrug efflux pump systems, which are often observed behind multidrug resistance. Additionally, the co-occurrence of resistance to specific antibiotics may be explained by the localization of certain genes within the same gene cassette. Special emphasis should be placed on the carriage of these genes on plasmids, phages, or as mobile genetic elements, as these are the primary drivers of horizontal gene transfer.

## Figures and Tables

**Table 1:** Statistical analysis of the relationship between the sample source and the level of resistance.

Antibiotics	Respiratory–Cloaca Comparation
	*p*-Values
Ceftriaxone	0.0327 *
Imipenem	0.0613
Doxycycline	0.6158
^1^ Potentiated sulphonamide	0.3223
^2^ Amoxicillin-clavulanic acid	0.3349
Florfenicol	0.5057
Enrofloxacin	0.7919
Amoxicillin	0.1864
Neomycin	0.6312
Spectinomycin	0.0494 *
Colistin	0.7426

* Significant difference (*p* < 0.05); ^1^ trimetoprime-sulphametoxazole 1:19 ration; ^2^ 1:2 ratio.

**Table 2:** Statistical analysis of resistance by utilization type.

Antibiotics	Laying–Meat	Laying–Breeding	Meat–Breeding
	*p*-Values		
Ceftriaxone	0.0900 *	0.9748	0.2026
Imipenem	0.5109	0.0196 *	0.0003 *
Doxycycline	0.6721	0.1697	0.4798
^1^ Potentiated sulphonamide	0.2893	0.0328 *	0.3857
^2^ Amoxicillin-clavulanic acid	0.0003 *	0.0185 *	>0.0001 *
Florfenicol	0.8774	0.0750 *	0.0148 *
Enrofloxacin	0.0209 *	0.1528	0.8835
Amoxicillin	>0.0001 *	0.1982	>0.0001 *
Neomycin	0.1641	0.9652	0.1257
Spectinomycin	0.0002 *	0.7831	0.0095 *
Colistin	0.4881	0.0008 *	0.0116 *

* Significant difference (*p* < 0.05); ^1^ trimetoprime-sulphametoxazole 1:19 ration; ^2^ 1:2 ratio.

**Table 3:** Statistical analysis of resistance by age group.

Antibiotics	^3^ Adult-^4^ Young
	*p*-Values
Ceftriaxone	0.0208 *
Imipenem	0.0045 *
Doxycycline	0.9387
^1^ Potentiated sulphonamide	0.7933
^2^ Amoxicillin-clavulanic acid	>0.0001 *
Florfenicol	0.0656
Enrofloxacin	0.0450 *
Amoxicillin	>0.0001 *
Neomycin	0.0240 *
Spectinomycin	>0.0001 *
Colistin	0.4138

* Significant difference (*p* < 0.05); ^1^ trimetoprime-sulphametoxazole 1:19 ration; ^2^ 1:2 ratio; ^3^ younger than 6 weeks; ^4^ older than 6 weeks.

**Table 4:** Statistical analysis of the relationship between herd size and resistance levels.

Antibiotics	Small-Medium	Small-Big	Medium-Big
	*p*-Values		
Ceftriaxone	0.4189	0.5839	0.1065
Imipenem	0.9788	0.9851	0.9999
Doxycycline	0.9569	0.9768	0.8985
^1^ Potentiated sulphonamide	0.3271	0.9377	0.2620
^2^ Amoxicillin-clavulanic acid	0.3347	>0.0001 *	>0.0001 *
Florfenicol	0.4135	0.0703 *	0.0044 *
Enrofloxacin	0.1308	0.0004 *	0.1215
Amoxicillin	0.5899	>0.0001 *	>0.0001 *
Neomycin	0.7628	0.2728	0.6761
Spectinomycin	0.2136	>0.0001 *	0.0060 *
Colistin	0.0027 *	>0.0001 *	0.3663

* Significant difference (*p* < 0.05); ^1^ trimetoprime-sulphametoxazole 1:19 ration; ^2^ 1:2 ratio; small—5001–50,000 birds; medium—50,001–100,000 birds; big—>100,001 birds.

**Table 5:** **Frequency table of the minimum inhibitory concentration (MIC) values for breakpoint agents in house chicken-derived ***Escherichia coli*** samples (n = 410**). In the top row for each agent, the number of occurrences is shown, while the percentage is presented in the bottom row. The vertical red line indicates the breakpoint.

Antibiotic	^1^ BP *	0.001	0.002	0.004	0.008	0.016	0.03	0.06	0.125	0.25	0.5	1	2	4	8	16	32	64	128	256	512	1024	MIC_50_	MIC_90_	^2^ ECOFF
	µg/mL																						µg/mL		
Enrofloxacin	^1^ 2	23	0	7	5	25	28	15	8	14	30	38	29	31	43	53	34	15	6	1	3	2	2	32	0.125
		5.6%	0.0%	1.7%	1.2%	6.1%	6.8%	3.7%	2.0%	3.4%	7.3%	9.3%	7.1%	7.6%	10.5%	12.9%	8.3%	3.7%	1.5%	0.2%	0.7%	0.5%			
Colistin	2	14	1	4	3	10	13	40	65	70	44	20	11	6	2	5	9	8	11	2	29	43	0.25	1024	2
		3.4%	0.2%	1.0%	0.7%	2.4%	3.2%	9.8%	15.9%	17.1%	10.7%	4.9%	2.7%	1.5%	0.5%	1.2%	2.2%	2.0%	2.7%	0.5%	7.1%	10.5%			
Ceftriaxone	^1^ 4	11	0	4	7	27	73	95	31	24	19	14	7	4	10	14	7	8	8	15	12	20	0.063	256	0.125
		2.7%	0.0%	1.0%	1.7%	6.6%	17.8%	23.2%	7.6%	5.9%	4.6%	3.4%	1.7%	1.0%	2.4%	3.4%	1.7%	2.0%	2.0%	3.7%	2.9%	4.9%			
Imipenem	^1^ 4	8	1	2	5	12	16	16	78	62	80	39	30	41	16	3	1						0.25	4	0.5
		2.0%	0.2%	0.5%	1.2%	2.9%	3.9%	3.9%	19.0%	15.1%	19.5%	9.5%	7.3%	10.0%	3.9%	0.7%	0.2%								
^3^ Potentiated sulphonamide	^1^ 4											3	61	71	41	40	17	12	16	13	38	98	1	64	0.5
												0.7%	14.9%	17.3%	10.0%	9.8%	4.1%	2.9%	3.9%	3.2%	9.3%	23.9%			
Doxycycline	^1^ 16										11	40	86	64	26	52	62	49	10	7	3		8	64	8
											2.7%	9.8%	21.0%	15.6%	6.3%	12.7%	15.1%	12.0%	2.4%	1.7%	0.7%				
Florfenicol	^1^ 16											1	16	48	88	111	54	48	3	18	17	6	16	128	16
												0.2%	3.9%	11.7%	21.5%	27.1%	13.2%	11.7%	0.7%	4.4%	4.1%	1.5%			
Neomycin	32											6	47	61	14	31	71	100	40	21	15	4	32	128	8
												1.5%	11.5%	14.9%	3.4%	7.6%	17.3%	24.4%	9.8%	5.1%	3.7%	1.0%			
Amoxicillin	^1^ 32					1	0	1	4	15	11	8	20	53	50	17	10	14	33	32	54	87	128	1024	8
						0.2%	0.0%	0.2%	1.0%	3.7%	2.7%	2.0%	4.9%	12.9%	12.2%	4.1%	2.4%	3.4%	8.0%	7.8%	13.2%	21.2%			
^4^ Amoxicillin-clavulanic acid	32							2	6	15	8	2	20	74	109	83	29	27	26	9			4	64	8
								0.5%	1.5%	3.7%	2.0%	0.5%	4.9%	18.0%	26.6%	20.2%	7.1%	6.6%	6.3%	2.2%					
Spectinomycin	128													1	9	56	88	120	61	27	29	19	64	512	64
														0.2%	2.2%	13.7%	21.5%	29.3%	14.9%	6.6%	7.1%	4.6%			

* BP—breakpoint; ^1^ Clinical Laboratory Standard Institute (CLSI); ^2^ Epidemiological cut-off value (EUCAST); ^3^ trimetophrime-sulphamethoxazole 1:19 ratio ^4^; 2:1 ratio.

## Data Availability

The data presented in this study are available from the corresponding author upon reasonable request.

## Supplementary Materials

The following supporting information can be downloaded at: https://www.mdpi.com/article/10.3390/antibiotics13121175/s1. Table S1: Frequency table of the minimum inhibitory concentration (MIC) values (µg/mL) for agents without breakpoints in *Escherichi coli* samples derived from chickens (n = 410).
