# Supplementary material for: Antimicrobial Susceptibility Profiles of Commensal Escherichia coli Isolates from Chickens in Hungarian Poultry Farms Between 2022 and 2023
Source: Antibiotics (Basel). 2024 Dec 4;13(12):1175. doi: 10.3390/antibiotics13121175 (PMC11672764; doi:10.3390/antibiotics13121175)
Supplement: Supplementary file 1 [file antibiotics-13-01175-s001.zip › antibiotics-3333343-supplementary.pdf]

Supplementary Table S1 Frequency table of the minimum inhibitory concentration (MIC) values (µg/mL) for agents without breakpoints in *Escherichi coli* samples derived from chickens (n=410). The top row for each agent shows the count, while the bottom row shows the percentage.

Antibiotic	0.001	0.002	0.004	0.008	0.016	0.03	0.06	0.125	0.25	0.5	1	2	4	8	16	32	64	128	256	512	1024	MIC_{50} µg/mL	MIC_{90} µg/mL
**Tilozin**											15	3	1	0	0	0	6	11	86	164	124	512	1024
											3.7%	0.7%	0.2%	0.0%	0.0%	0.0%	1.5%	2.7%	21.0%	40.0%	30.2%		
**Tiamulin**											1	2	5	1	2	11	46	102	115	43	82	256	1024
											0.2%	0.5%	1.2%	0.2%	0.5%	2.7%	11.2%	24.9%	28.0%	10.5%	20.0%		
**Lincomycin**												1	2	3	8	2	6	6	9	131	242	1024	1024
												0.2%	0.5%	0.7%	2.0%	0.5%	1.5%	1.5%	2.2%	32.0%	59.0%		
**Vancomycin**																	32	12	186	149	31	256	512
																	7.8%	2.9%	45.4%	36.3%	7.6%		
